# Epidemiological study of HPV in oral mucosa through PCR

**DOI:** 10.1590/S1808-86942012000400013

**Published:** 2015-10-20

**Authors:** Willys Tristão, Rodrigo Metzker Pereira Ribeiro, Camila Andrea de Oliveira, Julio Cesar Betiol, Jussara de Sousa Ribeiro Bettini

**Affiliations:** 1 Department of Obstetrics and Gynecology, School of Medicine - University of São Paulo (Master Degree student); 2 Graduate program in Biosciences - Fundaçã o Hermínio Ometto - UNIARARAS (Advisor); 3 Graduate program in Biosciences - Fundaçã o Hermínio Ometto - UNIARARAS (Co-Advisor); 4 B.A. In Pharmacy (Scientific Initiation Student - bound to the Graduate Program in Biomedical Sciences)

**Keywords:** mouth mucosa, papillomavirus infections, polymerase chain reaction

## Abstract

The Human Papillomavirus (HPV) belongs to the *Papillomaviridae* family and has a capsid and a single DNA strand. Its infection occurs mainly through sexual intercourse, having an important tropism for skin and mucosal cells.

**Aim**: To evaluate the HPV presence in normal oral mucosa of asymptomatic subjects and; in parallel, to correlate social behavioral habits with the virus.

**Materials and Methods**: Contemporary cohort cross-sectional study. The HPV was found by PCR, using general primers MY09/11 in 125 oral mucosa samples submitted to DNA extraction and PCR to search for the beta-globin gene in order to assess the quality of the extracted DNA. In parallel, we carried out a study of behavioral issues associated with the patients.

**Results**: All the samples had a positive diagnosis of the beta-hemoglobin gene. HPV was diagnosed in 23.2% of the samples analyzed.

**Conclusion**: The virus was present in 29 of the 125 patients, without them having any clinical-pathological manifestation associated with the HPV. As to the social behavior of the patients, we concluded that oral sex is statistically correlated to the virus, and besides the HPV has been statistically more present in female patients.

## INTRODUCTION

The human papillomavirus (HPV) belongs to the Papillomaviridae family. They are small, epitheliotropic and have about 55 mm in diameter. Their genome is made up of 7,200 to 8,000 base pairs with molecular weights of 5.2 × 106 daltons. The HPV is made up of a capsid with 72 capsomeres of icosahedral structures, with a lipoprotein envelope and one single circular double DNA molecule^1^. Its genome is divided into E (Early - coding the proteins which are initially produced) and L (Late - codes proteins which will be produced after those from the E region). These two regions represent 45% and 40% of the viral genome, respectively, and have the open reading frames (ORFS) E1, E2, E4, E5, E6, E7, L1 and L2. It is known that the E1 gene is involved in viral DNA replication and maintenance; E2 regulates viral transcription and E4 codes a protein which destroys cytoplasmic keratin, producing the koilocytosis halo image. It is believed that E5, E6 and E7 are involved in cell transformation and degradation, while L1 and L2 code the viral capsid proteins. Between the E and L regions, there is the non-coding region called LCR (long control region) or URR (upstream regulatory region), representing 15% of the viral genome involved in the control of viral gene expression^2^.

HPV infection is basically through sexual intercourse. As such, both men and women are involved in the epidemiological chain of the infection and are capable, at the same time, of being asymptomatic carriers, transmitters and victims of HPV infections. In these regards, the risk factors are clearly associated with the individual's sexual behavior. The most important are: starting sexual intercourse at an early age, having a large number of sexual partners during one's life and having sexual contact with high-risk individuals (i.e. men having frequent contact with female prostitutes; in the case of women, having intercourse with men who have multiple sexual partners)^3^.

HPV infects epithelial and mucosal cells, which can cause a number of hyperplastic lesions. Therefore, this virus can be mucosotropic, which infect the oral, respiratory and genital mucosae; and cutaneotropic, found in immune-competent individuals and in those with verruciform epidermoplasias^4^. Since the HPV infects only the epithelial layer, not going beyond the basal membrane, the primary immune exposure must happen by means of mechanisms present in this layer. The infected cell membrane expresses only the E5 protein, and this small quantity of surface viral antigens may damp the immune response. E5 binds and inactivates the protein that is needed to process the antigens. Since the epithelial cell is not a good antigen-presenting cell, the HPV remains inside it without being recognized by the immune system. Because the HPV does not cause host cell lysis or death, the virus remains isolated from contact with immune system cells, which would trigger the recognition process^5^.

The viral infection may cause localized clinical, subclinical or latent lesions. In general, the HPV follows the classic viral productive cycle: adsorption, penetration, transcription, translation, DNA replication and maturation. However, in some cases, such process does not happen in a complete way, since the virus may integrate the genome of the host cell and cause carcinogenesis. In benign lesions, the virus is in its circular form, called episomal - not integrated to the host cell genome, and in a large number of copies. In malignant lesions, it is integrated to the host cell genome. Notwithstanding, it is possible to find episomal forms in the malignant cells and, once integrated, the virus cannot be reverted to its episomal form^6^.

Viral types of high and low malignance risks differentiate according to the transformation capacity of E6 and E7 oncoproteins, coded by genes E6 and E7. E6 and E7 oncoproteins bind to p53 and pRB proteins, respectively, regulators of the cell cycle, considered cancer suppression. This phenomenon unblocks the cell cycle and causes genetic instability, which causes additional genetic changes, causing cancer by preventing apoptosis, leading to cell immortalization. This process is found only in high risk virus, and it is not seen in their low risk counterparts^7^.

It is estimated that in Brazil there are from 500 thousand to 1 million new cases per year of HPV infection, while 80 thousand cases of AIDS, 200 thousand to 500 thousand cases of herpes, 100 thousand cases of syphilis and 800 thousand cases of gonorrhea are registered^8^. The presence of anogenital HPV 6/11 and 16/18 in the oral mucosa could mean orogenital transmission, which would make this virus an important cofactor in the development of oral cancer, as it is considered in the uterine cervix^9^. The HPV prevalence in the oral mucosa, macroscopically normal, varies substantially in the literature: between 0 to 81.1%, with a mean of 10%. The infections are not always seen macroscopically. Thus, the HPV infection may be classified into: (a) latent, which may only be diagnosed by means of molecular biology assays; (b) subclinical, which does not bear clinical symptoms, but there are subtle changes which can be detected by diagnostic methods such as peniscopy, colpocytology, colposcopy and/or biopsy; (c) clinical, in which there are evident lesions upon clinical exam. From the molecular viewpoint, it is not known how an HPV infection remains latent, and anther develops macroscopic lesion of intense viral replication^10^. Progresses in the field of molecular biology and genetics have contributed substantially to study these viruses. Of all HPV DNA detection techniques, the polymerase chain reaction (PCR) is the most sensitive^11^.

The human papillomavirus (HPV) stands out as one of the most common sexually transmitted diseases (STD) in the world. Every year, the Brazilian Health Department registers 137 thousand new cases in the country. Since the viral diagnosis can be made still in the latent phase of the infection, it is possible to follow the patient up in order to educate him/her about proper treatment for the lesions or alterations which may arise because of the viral infection progression. Thus, our study aims at establishing the HPV prevalence in the oral mucosa of patients who do not have any type of lesion and, concurrently, assess whether sexual and social habits, such as sexual activity, having a fixed partner, intercourse with prostitutes, oral sex practice, use of condoms and smoking, are associated with the virus.

## MATERIALS AND METHODS

This study has been previously submitted to the appreciation and approval of the Ethics in Research Committee of the Institution, under protocol number: 840/2007.

### Sample Selection

We randomly collected 125 samples of oral mucosa swabs, from men and women, whom were told about the study, filled out the questionnaire with behavioral questions and signed the informed consent form, accepting to participate in the study. For sample collection purposes, we used the “Campos da paz” brush and the material was stored in *Eppendorf®*, tubes with 0.9% saline. They were then frozen for preservation until the extraction.

### Behavioral Questionnaire

The behavioral questionnaire was administrated after prior explanation about its importance in the study and that its data would be treated with the uttermost confidentiality (Chart 1).

**Chart 1:** Regarding the Behavioral Questionnaire.

Behavioral questionnaire
ID number:________
Skin color:________
Age:__Gender: (_) F or (_) M
Marital Status:________
Occupation: Program:________
Has already had sex? (_) Yes (_) No
Age of the first sexual intercourse:________
Fixed partners: (_) Yes (_)No
Number of partners during 1 year:
(_) 1 to 3 (_) 4 to 7 (_) 10 or more
Have you had sexual intercourse with prostitutes?
Yes (_) No (_)
Do you practice Oral Sex: (_) Yes (_) No
Do you use condoms: (_) Yes (_) No
How often do you use condoms:
(_) in all intercourses (_) Sometimes (_) Never
Do you have, or have you had lesions in your mouth: (_) Yes (_) No
Frequency: (_) Frequently (_) Occasionally (_) Rarely
Do you have Herpes or HPV?: (_) Yes (_) No (_) Unknown
Does your partner have Herpes or HPV? (_) Yes (_) No (_) Unknown
Have you ever had any STD?________
Smoker (_) Yes (_) No
Do you use medications (immune suppressors)? (_) Yes (_) No

### Statistical Analysis

The Fisher's exact test was used as statistical tool in order to check whether the HPV prevalence would be statistically associated to sex and the patients' life habits. A *p*-value below 0.05 proves correlation. We also obtained confidence interval (CI) values, set at 95% and Odds Ratio. Statistical data were produced by the SISA® (Simple Interactive Statistical Analysis) software.

### DNA Extraction

Initially, we stirred the samples in vortex (AP56 - Phoenix - Brazil), followed of the removal of 300 μl parts, which were transferred to another container. It was centrifuged and the supernatant was discarded. To the precipitate we added 600 μl of sodium hydroxide (NaOH) at 50 mM. We took it to hot water bath (MA159 - Marconi - Brazil) at 95°C for 5 minutes and immediately after we added 60 μl of 1 M tris-HCL. The material extracted was frozen.

### Polymerase Chain Reaction (PCR)

All the samples were submitted to PCR, using beta-globin to test the extraction method and avoid false negatives, since this gene is common to all human beings, thus showing up in all the samples.

To prepare the PCR mix for beta-globin, we used 1.5 mL of *Eppendorf®*. The reaction mix was made up of: 12.5 μl of deionized and autoclaved water, 2.5 μl of reaction buffer (10x - *Invitrogen*®), 1.0 μl of MgCl_2_ (50 mM - *Invitrogen®)*, 2.5 μl of dNTP (deoxynucleoside triphosphate) solution (2 mM - *Invitrogen*®), 1.0 μl of the pair of primers^12^ GH_2_0 (GAAGAGCCAAGGACAGGTAC)/PC04 (CAACTTCATCCACGTTCACC) at 10 pmol/μl (*Invitrogen®)*, 4 μl of DNA and 0.5 μl of Taq polymerase (5U/μl - *Invitrogen®)*, respectively, making up a total volume of 25 μl. In all amplified lots, we used negative controls (4 μl of water instead of DNA) and positive controls to detect possible contaminants during the reaction.

In order to prepare the PCR reaction mix for the HPV, we used 1.5 mL of *Eppendorf®*. The reaction mix is, respectively, made up of: 12.0 μl of autoclaved and deionized water, 2.5 μl of reaction buffer (10x - *Invitrogen*®), 1.5μl of MgCl_2_ (50mM - *Invitrogen®)*, 2.5 μl of dNTP (deoxynucleoside triphosphate) solution (2 mM - *Invitrogen*®), 1.0μl pair of general *primer*^2^ for HPV MY09 (GT CCM AAR GGA WAC TGA TC*)/MY11 (GCM CAG GGW CAT AAY AAT GG*) at 10 pmol/μl (*Invitrogen*®), 4 μl of DNA and 0.5 μl of Taq polymerase (5U/μl - *Invitrogen*®), making up a total volume of 25 μl. In all the analyses we used negative controls (4 μl of water instead of DNA) and positive controls to detect contaminations and act as patterns of viral presence.

Thermal cycles for the PCR reactions were carried out at the Peltier Thermal Cycler® (Biocycler Thermalcyclers), and the beta-globin amplification program was made up of: one initial cycle at 95°C for 5 minutes, 35 sequential cycles made up of three stages (denaturation at 94°C for 1 minute, hybridization at 60°C for 1 minute and synthesis at 72°C for 1 minute) and a final cycle at 72°C for 10 minutes. The PCR execution program for the general HPV primer was made up of: one initial cycle at 95°C for 5 minutes, 35 sequential cycles made up of three stages (denaturation at 94°C for 1 minute, hybridization at 40°C for 1 minute and synthesis at 72°C for 1 minute) and one final cycle at 72°C for 10 minutes.

The PCR products were submitted to electrophoresis in 1.5% agarose gel (0.75 g of agarose in 50 ml of TBE 1x and 5.0 μl of ethidium bromide buffer in a horizontal dish (Horizon 11-14® - Lifetechnologies - USA) under a potential difference of 100 volts during one hour. The gel was analyzed in a photo-Prep® transiluminator, at which 268 pb bands were considered positive for beta-globin. For the product arising from the amplification with the general primer for HPV, we utilized the 10% Bis-acrylamide gel electrophoresis technique (23.33 ml of Bis-Acrylamide; 4.9 ml of Glycerol; 7 ml of TBE 10x; 52.5 μl of Temed; 1050 μl of potassium persulphate and 34.1 ml of distilled water), which was taken to a potential difference of 120 volts for 3 hours in a vertical dish (Model V15-17® - Lifetechnologies - USA) and the gel dyed by the silver nitrate protocol^13^, in order to visualize the bands.

## RESULTS

**Figure 1:**
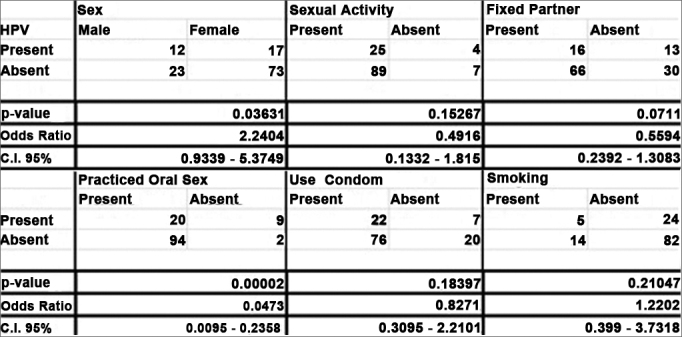
Fischer's Exact Test. (Statistically significant association when *p <* 0.05).

We found viral DNA in 23.2% (29/125) of the samples, and they were all positive for the beta-globin amplification gene, guaranteeing reliability to the experiment. As to the behavioral questionnaire, among the variables studied (Gender, Sexual Activity, Fixed Partner, Sexual Intercourse with Prostitutes, Oral Sex, Condom Use and Smoking), it was only the patient gender (OR = 2.2404, CI [0.9339 - 5.3749], *p* = 0.03631) and having oral sex (OR = 0.0473, CI [0.0095 - 0.2358], *p* = 0.0473) were statistically correlated with HPV (Figure 1).

Among the individuals investigated in the study, 37 (29.6%) volunteers were men, in the age range of 17-54 years and 88 (70.4%) were women, in the age range of 17-44 years. The age range between 18-21 predominated in both genders. Of the 29 positive HPV samples, 41.4% (12/29) were men and 58.6% (17/29) were women; 17.24% (5/29) were smokers; 24.13% (7/29) said they did not use condoms in all their sexual intercourses; 68.96% (20/29) stated they practiced oral sex; 13.79% (4/29) had not started their sexual life; 44.82% (13/29) did not have fixed partners, and 13.79% (4/29) had had sexual intercourse with prostitutes.

## DISCUSSION

The literature describes numerous cytopathic effects associated with HPV infection (koilocytosis, nucleus widening, megalocytosis, binucleation, and others), as well as intraepithelial lesions, which vary from low grade (LSIL) to high grade (HSIL), which may progress to squamous cell carcinoma (SCC)^14^. Notwithstanding, HPV may be asymptomatic - given that most infections are subclinical or latent. According to Xavier et al.^10^, the HPV prevalence in a macroscopically normal oral mucosa is very much variable in the literature: it varies between 0 and 81.1%, with a mean value of 10%. Castro & Bussoloti^15^ stated that detection rates may vary between 0 and 100%, even when using a more sensitive method, such as PCR, and mention that this large discrepancy in results may be caused by errors at the time of material harvesting, which may compromise the final results. In a larger proportion, when compared to our results, Peixoto^5^ had 100 patients without oral mucosa lesion in his cohort and diagnosed 81% viral prevalence; although, differently from our study, he did not find statistically significant association between having oral sex and HPV. Nonetheless, in agreement with our results, Cardesa & Nadal^16^ reported that oral sex is one of the main risk factors for the progression of the disease caused by the HPV. In regards to smoking and sex, Gilison et al.^17^ reported that HPV was more prevalent in men and smokers - their finding was statistically relevant, and they also reported a 6.9% prevalence of HPV in the general population of the United States of America - when they assessed a cohort with 5,579 individuals without prior selection. Kreimer et al.^18^, analyzed 1,688 samples from oral mucosae of healthy men - in agreement with our study - and did not find statistically significant association between the number of sex partners, use of condoms, as well as sexual activity and HPV in the oral mucosa. According to Castellsagué^3^, the use of condoms is a factor which may reduce the risks of HPV transmission; however, it does not guarantee total protection.

## CONCLUSIONS

The human papillomavirus could be found in 23.2% of the 125 cases studied. Since these patients do not bear any clinical-pathological manifestation associated with the virus, the HPV may be present in healthy oral mucosae in significant proportions. Although American cohort studies have reported higher HPV prevalence in the oral mucosae of men - among the patients included in the study, the women and/or those who have oral sex made up the risk group in which HPV was statistically more prevalent.

Although other life habits did not reach statistical significance, studies with more patients must be carried out, since factors such as smoking, not using condoms, having multiple sex partners and sexual activity are factors which increase the likelihood of transmission and/or infection by the HPV.
